# Bcl-2 family proteins as regulators of cancer cell invasion and metastasis: a review focusing on mitochondrial respiration and reactive oxygen species

**DOI:** 10.18632/oncotarget.6405

**Published:** 2015-11-26

**Authors:** Hong-Duck Um

**Affiliations:** ^1^ Division of Radiation Cancer Biology, Korea Institute of Radiological & Medical Sciences, Seoul, Korea

**Keywords:** Bcl-2 family, cancer invasion and metastasis, reactive oxygen species, mitochondrial respiration

## Abstract

Although Bcl-2 family proteins were originally identified as key regulators of apoptosis, an impressive body of evidence has shown that pro-survival members of the Bcl-2 family, including Bcl-2, Bcl-X_L_, and Bcl-w, can also promote cell migration, invasion, and cancer metastasis. Interestingly, cell invasion was recently found to be suppressed by multidomain pro-apoptotic members of the Bcl-2 family, such as Bax and Bak. While the mechanisms underlying these new functions of Bcl-2 proteins are just beginning to be studied, reactive oxygen species (ROS) have emerged as inducers of cell invasion and the production of ROS from mitochondrial respiration is known to be promoted and suppressed by the pro-survival and multidomain pro-apoptotic Bcl-2 family members, respectively. Here, I review the evidence supporting the ability of Bcl-2 proteins to regulate cancer cell invasion and metastasis, and discuss our current understanding of their underlying mechanisms, with a particular focus on mitochondrial respiration and ROS, which could have implications for the development of strategies to overcome tumor progression.

## INTRODUCTION

Bcl-2 family proteins are key regulators of cell death that can either suppress or promote apoptosis [1-3]. The pro-survival subfamily includes Bcl-2, Bcl-X_L_, Bcl-w, Mcl-1, and A1, whereas the pro-apoptotic subfamily is classified into the multidomain group (Bax, Bak, and Bok) and the BH3-only group (Bid, Bim, Bad, and others), based on the number of BH domains they possess. The mechanisms through which Bcl-2 proteins regulate cell death have been extensively characterized for more than two decades. Briefly, pro-survival Bcl-2 family members support cellular viability by binding to multidomain pro-apoptotic Bcl-2 family members and inhibiting their apoptotic activities. This inhibitory interaction is disrupted by BH3-only members, which are activated upon apoptotic stimulation of cells. Multidomain pro-apoptotic members then attack the mitochondria, inducing mitochondrial outer membrane permeability and apoptosis. Based on this theory, multidomain pro-apoptotic members act as executioners of apoptosis, whereas pro-survival and BH3-only members are inhibitors and sensors/initiators of apoptosis, respectively.

The pro-survival and pro-apoptotic members of the Bcl-2 family are frequently up- and down-regulated, respectively, in numerous types of cancer cells. Such differential expression is believed to contribute to tumorigenesis and the resistance of cancer cells to anticancer treatments [4]. However, beginning in the late 1990s [5-12], researchers have reported that the functions of Bcl-2 proteins are not confined to cell death control, and at least some Bcl-2 proteins can also regulate cell migration, invasion, and cancer metastasis. This has been further supported by an impressive body of subsequent studies analyzing the functions of Bcl-2 proteins in cellular and animal models and evaluating their clinical relevance using patient samples. While the mechanisms underlying these non-apoptotic functions of Bcl-2 proteins are still poorly understood, reactive oxygen species (ROS) have emerged as key inducers of cell invasion. Moreover, increasing evidence suggests that pro-survival Bcl-2 family members promote cell invasion by facilitating ROS production from mitochondrial respiration, whereas multidomain pro-apoptotic members suppress cell invasion by decreasing ROS production. In this review, I attempt to provide an overview of the function of Bcl-2 proteins in the regulation of cancer cell invasion and metastasis. I also discuss our current understanding of the underlying mechanisms with a particular focus on ROS and mitochondrial respiration.

## PRO-SURVIVAL BCL-2 FAMILY MEMBERS PROMOTE CELL MIGRATION, INVASION, AND TUMOR METASTASIS

### Studies at the cellular level

The overexpression of Bcl-2 in various cancer cell types, including glioma [6, 13], neuroblasotoma [14], melanoma [15], squamous carcinoma [16], breast [5], lung [17], and colorectal cancer cells [18], increases the migratory and invasive potentials of these cells. Such effects have also been observed following overexpression of Bcl-X_L_ [18-22], Bcl-w [22-26], or Mcl-1 [18] in glioma [19, 22], lung [20, 25], colorectal [18, 21], and gastric cancer cells [23, 24, 26]. Cellular invasiveness is consistently reduced when Bcl-2 [18], Bcl-X_L_ [18, 21, 27], Bcl-w [26], or Mcl-1 [18] expression is reduced, indicating that these pro-survival members promote cell migration and invasion. These functions have also been confirmed in normal cells; overexpression of Bcl-2 in vascular smooth muscle cells [28] and Bcl-w in mouse embryonic fibroblasts (MEFs) [22] enhances cellular invasiveness. However, Bcl-2 overexpression is not always sufficient for inducing its pro-invasive effects, and additional treatments, such as co-expression of Bcl-2 with C-Myc [28], N-Myc [14], or Twist1 [29] and exposure to hypoxic conditions [15], may be necessary in certain cases. Similarly, knockout of Bcl-X_L_ in pancreatic cancer cells does not significantly influence basal invasiveness, but prevents cell invasion induced by treatment with CoCl_2_ [27]. Therefore, the pro-invasive activity of pro-survival Bcl-2 family members appears to vary depending on the cell type and environment (Table 1).

**Table 1:** Regulation of cell migration and invasion by Bcl-2 family proteins

Protein	Cell type(s)	Function(s)	References
Bcl-2	Breast cancer and glioma cells	Promotes cell invasion by inducing MMP-2, MMP-9, and uPA	[5, 6]
	Glioma cells	Promotes cell invasion by inducing MMPs via the furin/TGFβ pathway	[13]
	Lung cancer cells	Promotes cell invasion by inducing MMP-2 via AP-1	[17]
	Squamous carcinoma cells	Promotes cell migration and invasion by inducing MMP-9 via the N-cadherin/FGF receptor/ERK pathway	[16]
	Colorectal cancer cells	Promotes cell migration and invasion	[18]
	Neuroblastoma cells	Bcl-2/N-Myc co-expression promotes cell invasion by inducing MMP-2	[14]
	Vascular smooth muscle cells	Bcl-2/c-Myc co-expression promotes cell migration and invasion by inducing MMP-2	[28]
	Hepatocarcinoma cells	Bcl-2/Twist co-expression promotes cell migration and invasion	[29]
	Melanoma cells	Promote cell invasion by inducing MMP-2 under hypoxic conditions	[15]
Bcl-X_L_	Glioma cells	Promotes cell invasion by inducing MMP-2 via AP-1	[19]
	Lung cancer cells	Promotes cell invasion by inducing the PI3K/p38/Akt/MMP-2 pathway	[20]
	Colorectal cancer cells	Promotes cell migration and invasion	[18, 21]
	Pancreatic cells	Promotes cell invasion under chemically induced hypoxic conditions	[27]
Bcl-w	Gastric cancer cells	Promotes cell migration and invasion by inducing PI3K, Akt, Sp1, MMP-2, uPA, FAK	[23, 24, 26]
	Lung cancer cells	Promotes cell invasion by inducing the Src-EGFR pathway	[44]
Bcl-w, Bcl-X_L_	Glioma, lung cancer cells, and MEFs	Promote cell invasion by inducing ROS via complex-I	[22, 25]
Mcl-1	Colon cancer cells	Promotes cell migration and invasion	[18]
Bax, Bak	Glioma, lung cancer cells, and MEFs	Suppress cell invasion by binding to complex-I and inhibiting ROS production	[22, 25]

### Studies using animal models

Consistent with this pro-invasive activity, studies using mouse models have shown that pro-survival members of the Bcl-2 family can promote tumor metastasis. In a mouse pancreatic tumor model that was elegantly established by expressing an oncogene (SV40 T antigen) and a docking molecule for the virus in pancreatic islet β cells, additional delivery of the gene encoding Bcl-X_L_ into the cells using a viral vector was shown to promote the formation of islet tumors with invasive properties and enhance pancreatic lymph node metastasis [30]. Consistent with this, β-cell-specific knockout of Bcl-X_L_ in the same model was shown to decrease the incidence of invasive carcinoma and increase that of encapsulated, non-invasive tumors [27], supporting the hypothesis that Bcl-X_L_ promotes the invasion and metastasis of pancreatic tumors *in vivo*. Studies using a breast cancer model in which control and Bcl-X_L_-overexpressing breast cancer cells were transplanted into mouse intra-mammary fat pads revealed the ability of Bcl-X_L_ to accelerate the metastasis of breast cancer to the lymph nodes [31], lungs [32], and various other organs [33]. Another study also reported the ability of Bcl-2 to accelerate lung metastasis in a similar breast cancer model [34]. Moreover, when Bcl-2-overexpressing tumor cells were injected into the footpad [17], leg muscle [5], or tail vein of mice [5, 16], their pulmonary metastasis was enhanced compared with that in control tumor cells. Consistent with this, overexpression of Bcl-X_L_ and Bcl-w facilitates the infiltrative growth of glioma cells [19] and the intravasation of lung cancer cells [25], respectively, in xenograft tumor models. Overall, these reports support the ability of pro-survival Bcl-2 family members to promote cancer cell infiltration, intravasation, and metastasis *in vivo*.

### Studies analyzing patient samples

Pathological analyses of patient samples have supported the clinical relevance of such new functions of pro-survival members of the Bcl-2 family. For example, Bcl-2 expression in cancer cells is associated with liver metastasis in colorectal cancer [8], lymphovascular invasion of breast cancer cells [35], and nodal metastasis and invasion in laryngeal squamous cell carcinoma [36]. Moreover, Bcl-2 up-regulation is particularly obvious during the progression from pre-invasive lesions to invasive carcinoma in lung cancer samples [9]. Studies analyzing Bcl-X_L_ expression patterns have also demonstrated the up-regulation of this protein in invasive and metastatic cancer cell populations and the correlation of Bcl-X_L_ expression with the invasion (vascular or stromal invasion) and metastasis (lymph node or distal metastasis) of retinoblastoma [37], breast cancer [10, 38], colon cancer [39], tongue cancer [40], and hepatocellular carcinoma cells [41]. Likewise, Bcl-w up-regulation is significantly associated with infiltrative types of gastric cancer [42] and invading populations, as opposed to tumor cores, in glioma cells [43].

## PRO-SURVIVAL BCL-2 FAMILY MEMBERS STIMULATE A VARIETY OF SIGNALING MOLECULES THAT SUPPORT CELL MIGRATION AND INVASION

**Figure 1:**
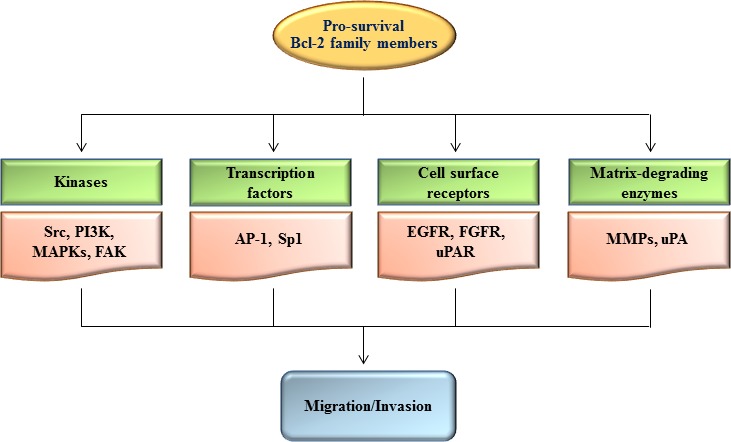
Regulation of cell migration and invasion by pro-survival Bcl-2 family members Up-regulation of pro-survival Bcl-2 family members results in stimulation of diverse sets of signaling components such as kinases, transcription factors, cell surface receptors, and matrix-degrading enzymes, leading to promotion of cell migration and invasion. The hierarchical relationship and importance of each signaling component may vary depending on the experimental conditions.

Ample evidence indicates that pro-survival Bcl-2 proteins activate cellular signaling pathways involved in cell migration and invasion. A series of studies performed using glioma [22], gastric cancer [23, 24], and lung cancer cells [25, 44] showed that Bcl-w overexpression stimulates the invasion pathway involving Src, epidermal growth factor receptor (EGFR), phosphoinositide 3-kinase (PI3K), Akt, Sp1, matrix metalloproteinase (MMP)-2, urokinase-type plasminogen activator (uPA), and focal adhesion kinase (FAK). Similarly, overexpression of Bcl-X_L_ in lung cancer cells increases PI3K and p38 mitogen-activated protein kinase (MAPK) activities, which subsequently induce MMP-2 expression via Akt [20]. Other groups have reported that AP-1 mediates Bcl-X_L_-induced MMP-2 expression in glioma cells [19]. Moreover, Bcl-2 overexpression in glioma [6, 13], neuroblastoma [14], melanoma [15], breast cancer [5], lung cancer [17], and vascular smooth muscle cells [28] increases the expression and activity of MMP-2, MMP-9, and uPA. The induction of MMP-2 and/or MMP-9 by Bcl-2 overexpression is mediated by AP-1 in lung cancer cells [17], furin and TGF-β in glioma cells [13], and the N-cadherin/FGF receptor/ERK pathway in squamous carcinoma cells [16]. Overexpression of Bcl-2 in melanoma cells also increases uPA receptor expression via ERK and Sp1 under hypoxic conditions [45]. Collectively, these studies indicate that pro-survival Bcl-2 proteins promote cell migration and invasion by increasing the activities of various sets of signaling molecules and matrix-degrading enzymes depending on experimental conditions (Table 1 and Figure 1).

## MULTIDOMAIN PRO-APOPTOTIC BCL-2 FAMILY MEMBERS SUPPRESS CELL INVASION

Compared with pro-survival members of the Bcl-2 family, pro-apoptotic members have received far less attention in studies of their ability to regulate cellular invasiveness, particularly for BH3-only members. However, my group has recently shown that multidomain pro-apoptotic members suppress cell invasion by inhibiting the PI3K/Akt/MMP-2 pathway [22, 25]. Specifically, knockdown of Bax and Bak in glioma, colon, or lung cancer cells promotes cell invasion by stimulating the PI3K/Akt/MMP-2 pathway, and Bax-knockout MEFs are consistently more invasive than control MEFs (Table 1). The anti-invasive functions of Bax and Bak appear to be clinically relevant, as supported by studies of various pathologies. For example, increased expression of Bax and Bak in oral squamous cell carcinoma is significantly correlated with the absence of vascular invasion [46]. Bax expression is also correlated with reduced lymph vessel invasion and reduced depth of invasion in colorectal carcinoma [7]. Moreover, Bax down-regulation is primarily observed in pre-invasive lesions and invasive carcinomas in patients with lung cancer [9] and is correlated with massive choridal invasion and pathological tumor-node-metastasis staging in retinoblastoma samples [37].

## BCL-2 PROTEINS REGULATE INVASION-RELATED PATHWAYS VIA ROS

How do Bcl-2 proteins regulate the cellular signaling processes? Most Bcl-2 proteins are functionally and physically associated with the mitochondria [1-3] which produce the majority of cellular ROS as byproducts of mitochondrial respiration. The mitochondrial respiratory chain is composed of four multimeric protein complexes, i.e., complex-I-IV. Among these complexes, complex-I and -III are major sources of ROS, producing O_2_^.−^, which is then converted to H_2_O_2_ [47, 48], a highly diffusible signaling molecule [49]. Mitochondrial ROS are thought to activate various signaling pathways involving PI3K, FAK, MAPKs, AP-1, MMPs, and uPA; these signaling pathways support cell migration and invasion [50]. Therefore, Bcl-2 proteins may regulate invasion-related pathways by modulating mitochondrial ROS production. Indeed, the ability of Bcl-2 proteins to regulate ROS production has been well recognized under apoptotic conditions, during which Bax promotes ROS production by inducing the mitochondrial permeability transition, a process that can be blocked by pro-survival Bcl-2 family members [51-53]. Bcl-2 proteins can also regulate ROS production in non-apoptotic healthy cells. However, the regulatory modes in this context are opposite those in apoptotic cells. Pro-survival members promote, rather than suppress, ROS production in healthy cells, as confirmed by overexpression of Bcl-2 [54-57], Bcl-X_L_ [22, 25, 56], Bcl-w [22, 25], and Mcl-1 [56] in various types of normal and cancer cells, including bacteria [58]. The levels of ROS induced by the overexpression of pro-survival members are relatively low, i.e., not sufficient for inducing cell death. However, inhibition of ROS induction using antioxidants or metabolic inhibitors prevents the ability of Bcl-w and Bcl-X_L_ to stimulate PI3K/Akt/MMP-2-dependent invasion pathways and promote cell invasion [22, 25], suggesting that these functions of pro-survival Bcl-2 family members can be mediated by ROS. In contrast, Bax and Bak suppress cell invasion by inhibiting ROS production; specifically, knockdown/knockout of Bax and Bak in MEFs and various cancer cell types increases basal ROS levels, and prevention of ROS accumulation using antioxidants or metabolic inhibitors abolishes the effects of Bax/Bak knockdown on the PI3K/Akt/MMP-2 pathway and cellular invasiveness [22, 25]. Thus, whereas pro-survival Bcl-2 family members stimulate pro-invasion signaling by increasing ROS production, multidomain pro-apoptotic members suppress pro-invasion signaling by inhibiting ROS production.

## BCL-2 PROTEINS REGULATE MITOCHONDRIAL RESPIRATION

Evidence suggests that pro-survival Bcl-2 proteins promote mitochondrial respiration. Bcl-2-induced ROS production in leukemia cells is accompanied by increases in cellular oxygen consumption, cytochrome c oxidase (COX)/complex-IV activity, and mitochondrial respiration [55, 59, 60]. Moreover, the overexpression of Bcl-2 [61] and Bcl-X_L_ [62] in osteosarcoma cells stimulates mitochondrial respiration, as shown by increases in oxygen consumption and the mitochondrial transmembrane potential (ΔΨm). Studies analyzing Bcl-X_L_-overexpressing [63] or Bcl-X_L_-knockout neurons [64] have also demonstrated the ability of Bcl-X_L_ to increase mitochondrial energetics and ATP levels. Moreover, the overexpression of Bcl-w or Bcl-X_L_ in lung cancer cells increases complex-I activity, ΔΨm, and cellular ATP levels [25]. These findings support the hypothesis that the pro-survival members of the Bcl-2 family promote ROS production by increasing mitochondrial respiratory activity. Indeed, rotenone, an inhibitor of complex-I, abolishes the ROS generation induced by Bcl-w and Bcl-X_L_ overexpression [22, 25].

In contrast, addition of recombinant Bax protein reduces ΔΨ_m_ and mitochondrial respiration in permeabilized astrocytes [65] and cardiomyocytes and in isolated rat heart mitochondria [66]. The ability of Bax to reduce ΔΨ_m_ and ROS production has also been shown in oligomycin-treated mouse sympathetic neurons [67]. Consistent with this, the knockdown of Bax and Bak in lung cancer cells increases complex-I activity, Ψ_m_, and ATP levels [25]. All of these studies support the ability of multidomain pro-apoptotic members to inhibit mitochondrial respiration. These findings also suggest that multidomain pro-apoptotic members suppress ROS production by inhibiting mitochondrial respiration, which is further supported by the ability of rotenone to prevent increases in ROS levels induced by Bax knockdown [25]. In summary, pro-survival and multidomain pro-apoptotic Bcl-2 proteins antagonistically regulate mitochondrial respiration and thus the production of respiratory ROS.

## MECHANISMS UNDERLYING THE ANTAGONISTIC REGULATION OF RESPIRATORY ROS PRODUCTION BY PRO-SURVIVAL AND MULTIDOMAIN PRO-APOPTOTIC BCL-2 FAMILY MEMBERS

Studies analyzing the hierarchical relationship between Bcl-w and Bax in the regulation of cellular invasiveness have shown that Bcl-w acts upstream of Bax to enhance cellular invasiveness [22]. Moreover, mutated Bcl-w and Bcl-X_L_ proteins that do not bind to Bax (i.e., Bcl-w^G94A^ and Bcl-X_L_^G138A^) fail to stimulate ROS production, cell invasion [22], and cancer cell intravasation in mouse models [25], supporting the notion that pro-survival Bcl-2 family members promote ROS production, cell invasion, and cancer metastasis by binding to multidomain pro-apoptotic members and blocking their inhibitory effects on ROS production.

**Figure 2:**
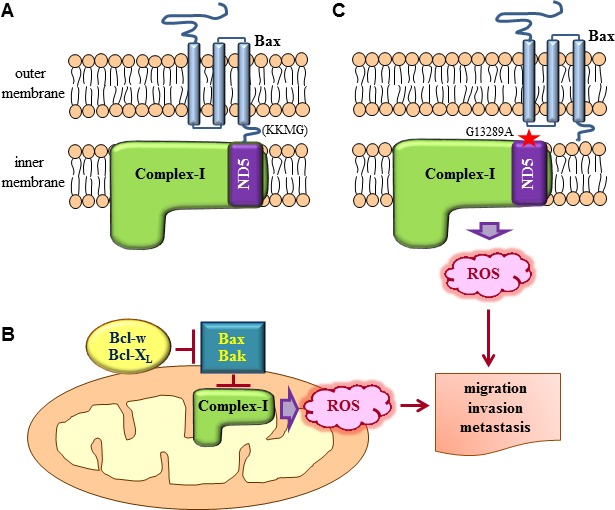
Regulation of complex-I-dependent ROS production by Bcl-2 family proteins **A.** Bax residing in the outer mitochondrial membrane in its tail-anchored form protrudes the four residues in its C-terminus (KKMG) into the intermembrane space. This topology allows the interaction of the C-terminal tail with subunits of complex-I in the inner mitochondrial membrane. ND5 is one such subunit that participates in the interaction. As a result of the interaction, the enzymatic activity and ROS-producing ability of complex-I are suppressed. Although not depicted here, Bak in the outer mitochondrial membrane also interacts with ND5 through its four C-terminal residues (FFKS). **B.** The interactions between Bax/Bak and complex-I are disrupted when Bcl-w and Bcl-X_L_ bind to Bax and Bak. Consequently, the inhibitory effects of Bax and Bak on complex-I are relieved, increasing complex-I activity and ROS production. **C.** Subunits of complex-I, particularly ND5, are frequently mutated in cancer. One such natural mutation of ND5 (ND5^G13289A^) prevents its interaction with Bax, promoting complex-I activity and ROS production. ROS generated by mutations in complex-I or the actions of pro-survival Bcl-2 family members then stimulate diverse signaling pathways, leading to the promotion of cancer cell migration, invasion, and metastasis.

Then, how do multidomain pro-apoptotic members suppress ROS production? Our finding that Bax and Bak interact with complex-I has provided insights into these mechanisms [25]. Human complex-I consists of 45 subunits, some of which reside in the inner mitochondrial membrane (membrane arm), or protrude into the mitochondrial matrix (matrix arm) [68, 69]. Bax and Bak can reside in the outer mitochondrial membrane in unstressed cells, with four residues of their C-terminal region (KKMG in Bax and FFKS in Bak) protruding into the intermembrane space [70-72]. Therefore, these C-terminal residues of Bax and Bak may interact with subunits of complex-I that are in its membrane arm, exposed to the intermembrane space. Bax and Bak indeed bind to the ND5 subunit of complex-I [25] (Figure 2A), which resides in the membrane arm and has a long C-terminal extension facing the intermembrane space [73]. These interactions are mediated by the four C-terminal residues of Bax and Bak; indeed, deletion of these residues abolishes the ability of Bax and Bak to interact with complex-I/ND5 and suppress ROS production and cell invasion [25]. This suggests that multidomain pro-apoptotic members suppress cell invasion by binding to complex-I and inhibiting its enzymatic and ROS-producing activity. In contrast, Bcl-w and Bcl-X_L_ do not bind to complex-I. However, the interactions of Bcl-w and Bcl-X_L_ with Bax and Bak facilitate the dissociation of Bax and Bak from complex-I, thereby relieving the inhibition of complex-I activity [25]. This model suggests that Bax and Bak are direct inhibitors of complex-I and that Bcl-w and Bcl-X_L_ stimulate complex-I by acting as inhibitors of Bax and Bak (Figure 2B), reminiscent of the mechanisms through which this family of proteins regulates apoptosis.

Most of the proteins involved in the mitochondrial respiratory chain are encoded by mitochondrial DNA, which is frequently mutated in various types of cancer [74]. Some of these mutations increase ROS production, suggesting that they may promote cancer cell invasion. Interestingly, approximately 20% of patients with lung cancer have mutations in the complex-I gene, and more than 80% of mutations in complex-I occur in the *ND5* gene [75]. Moreover, one such natural mutation in *ND5* (ND5^G13289A^) was shown to prevent the Bax/ND5 interaction [25], thereby increasing ROS production and cellular invasiveness [25, 75] (Figure 2C). These data support the clinical relevance of Bax/ND5 interactions in tumor progression.

## OTHER POSSIBLE MECHANISMS THROUGH WHICH PRO-SURVIVAL BCL-2 PROTEINS PROMOTE CELL INVASION

Complex-I may not be the only target through which Bcl-2 proteins regulate mitochondrial ROS production and cell invasion. As described above, overexpression of Bcl-2 in leukemia cells increases the overall rate of mitochondrial respiration and ROS production, accompanied by an increase in the localization of the Va and Vb subunits of COX in mitochondria and subsequent enhancement of COX activity [59, 60, 76]. These effects of Bcl-2 overexpression are thought to be mediated by the direct binding of Bcl-2 to COX Va [60]. Therefore, it is possible that Bcl-2 may contribute to cancer cell invasion and metastasis by targeting COX. However, it is not clear whether COX is a common target for other Bcl-2 proteins because COX Va has not been shown to interact with Bcl-X_L_, Bax, or Bak [60].

**Figure 3:**
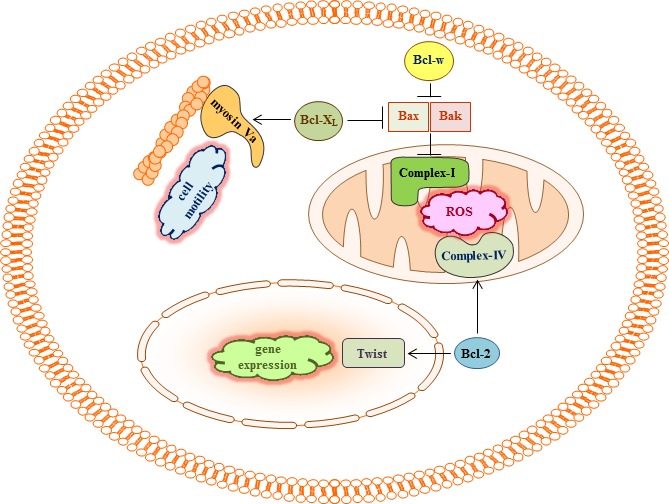
Bcl-2 proteins may regulate cell migration and invasion by binding to multiple targets In addition to the role of complex-I in cell migration and invasion, Bcl-2 has been reported to bind to Twist and COX Va, a subunit of complex-IV. Myosin Va was also shown to bind to Bcl-X_L_. Given the ability of Bcl-2 to facilitate the production of respiratory ROS, the ability of Twist to promote epithelial-mesenchymal transition and cell invasion, and the ability of myosin Va to regulate cell motility, such interactions may contribute to the pro-invasive activity of Bcl-2 and Bcl-X_L_. However, these possibilities have not been examined directly.

Pro-survival Bcl-2 proteins may also promote cell migration and invasion by interacting with proteins that are not directly involved in mitochondrial metabolism. For example, Bcl-2 binds to the transcription factor Twist1, and this interaction facilitates the nuclear import of Twist1, thereby promoting the transcription of a wide range of genes that can promote cell migration, invasion, and metastasis [29]. Moreover, Bcl-X_L_ directly binds to myosin Va [30]. Given the role of myosins in cell movement [77], the interaction of myosin Va with Bcl-X_L_ may influence cellular motility and invasiveness (Figure 3).

## CONCLUDING REMARKS

In this review, I have discussed evidence supporting the ability of Bcl-2 family proteins to regulate cancer cell invasion and metastasis and described the clinical relevance of these nontraditional functions of Bcl-2 proteins. Although Bcl-2 proteins may exert such functions via multiple mechanisms, this review focused on respiratory ROS because the mitochondria are major sites of Bcl-2 protein localization and because ROS can regulate various signaling pathways and cellular functions [49, 50]. Bcl-2 proteins are also thought to regulate other cellular functions, such as cell differentiation (epithelial-mesenchymal transition) [16, 20, 29, 78], senescence [79, 80], autophagy [81-83], and mitochondrial fusion and fission [84-86]. Therefore, the mitochondrial respiratory chain and ROS may also be involved in such diverse non-apoptotic functions of Bcl-2 proteins. Accordingly, identification of new binding partners of Bcl-2 proteins, analysis of their functions, and investigation of the possible ability of BH3-only members to regulate ROS production and cell invasion may provide new insights into the biology of Bcl-2 proteins and the regulation of cancer metabolism and metastasis.
